# Spatial clusters of extended-spectrum beta-lactamase-producing *Escherichia coli* causing community-onset bacteriuria due to repeat infections: cluster analysis from a large urban medical center, San Francisco, 2014–2020

**DOI:** 10.1186/s13756-023-01320-1

**Published:** 2023-10-20

**Authors:** Eva Raphael, Pushkar P. Inamdar, Cheyenne Belmont, Salma Shariff-Marco, Alison J. Huang, Henry F. Chambers

**Affiliations:** 1 Department of Epidemiology and Biostatistics, University of California, San Francisco, San Francisco, CA USA; 2 Department of Family and Community Medicine, University of California, San Francisco, San Francisco, CA USA; 3 Department of Medicine, University of California, San Francisco, San Francisco, CA USA; 4 Department of Urology, University of California, San Francisco, San Francisco, CA USA; 5 Global Health and Clinical Sciences, 550 16th Street, Box 0560, San Francisco, CA 94143 USA

## Abstract

**Background:**

Urinary tract infections caused by extended-spectrum beta-lactamase (ESBL)-producing *Escherichia coli* (ESBL-*E. coli*) may occur as outbreaks due to common-source exposures. Yet, it is currently unknown if they cluster geographically as would be expected as part of an outbreak.

**Methods:**

We collected electronic health record data on all patients living in San Francisco with culture-documented community-onset *E. coli* bacteriuria in a safety-net public healthcare system from January 2014 to March 2020 (diagnosed < 48 h after hospital admission or in outpatient clinical settings without a hospitalization in the past 90 days). We assessed the presence of spatial clusters of (1) ESBL-*E. coli* bacteriuria episodes, and (2) individuals with any ESBL-*E. coli* bacteriuria episode, with Global and Local Moran’s I. We evaluated differences in prevalence of bacteriuria recurrence by ESBL-production by Poisson regression.

**Results:**

Out of 4,304 unique individuals, we identified spatial clusters of ESBL-*E. coli* bacteriuria episodes (n = 461) compared to non-ESBL-*E. coli* bacteriuria episodes (n = 5477; Global Moran’s p < 0.001). Spatial clusters of individuals with any bacteriuria caused by ESBL-*E. coli* were not identified (p = 0.43). Bacteriuria recurrence was more likely to occur with ESBL-*E. coli* (odds ratio [OR] 2.78, 95% confidence interval [95% CI] 2.10, 3.66, p < 0.001), particularly after an initial ESBL-*E. coli* bacteriuria episode (OR 2.27, 95% CI 1.82, 2.83, p < 0.001).

**Conclusion:**

We found spatial clusters of ESBL-*E. coli* bacteriuria episodes. However, this was partly explained by clustering within individuals more than between individuals, as having an ESBL-*E. coli* bacteriuria was associated with recurrence with ESBL-*E. coli*. These findings may help better tailor clinical treatment of patients with recurrent urinary tract infections after an initial episode caused by ESBL-*E. coli*.

**Supplementary Information:**

The online version contains supplementary material available at 10.1186/s13756-023-01320-1.

## Key points

• In a large public healthcare system, from 2014 to 2020, we found spatial clusters of all extended-spectrum beta-lactamase-producing *Escherichia coli* (ESBL-*E. coli*) bacteriuria episodes, but not at the individual level. ESBL-*E. coli* was more likely to cause recurrent bacteriuria.

**Supplementary Information:**

The online version contains supplementary material available at 10.1186/s13756-023-01320-1.

## Introduction

In 2019, extended-spectrum beta-lactamase (ESBL)-producing Enterobacteriaceae were identified as “serious threat” pathogens in a Centers for Disease Control and Prevention (CDC) report on antimicrobial resistance [1] Since first emerging in skilled nursing facilities, the global public health threat of ESBL-producing *Escherichia coli* (ESBL-*E. coli*) infections has been well established with increasing prevalence in community settings [2–7]. Novel risk factors associated with such infections include international travel and consumption of food contaminated with ESBL-*E. coli* [8–15]. Thus, it is now hypothesized that such infections are due to common-source exposures and occur as outbreaks, defined as an increase in the number of observed disease cases above expected cases for that time and place [16]. Indeed, several studies have identified spatial clusters of drug-resistant Enterobacteriaceae infections in the community [17–20]. However, these prior studies have focused on samples from various clinical sources (i.e.; urine and blood), did not differentiate between repeat or recurrent episodes, or were restricted to short periods of time. Thus, it is currently unknown whether such spatial clusters are due to repeat infections in the same individuals, or if they represent multiple patients with bacteriuria caused by ESBL-*E. coli* in close geographic vicinity of one another which would suggest possible outbreaks.

Here, we sought to identify spatial clusters of community-onset bacteriuria episodes caused by ESBL-*E. coli* detected within a large urban public healthcare system from 2014 to 2020. We compared the frequency of recurrent bacteriuria by ESBL-production as potential clusters could be explained by bacteriuria recurrence in the same individual. Our goal was to provide new insight into spatial clustering of these infections in community settings that could inform public health surveillance efforts and help reduce drug-resistant pathogen transmission in the community.

## Methods

### Study design, settings, and population

This is an observational study focused on patients with culture-proven *E. coli* bacteriuria receiving care from the San Francisco Health Network and San Francisco General Hospital, a safety-net public healthcare system, from January 2014 to March 2020. This healthcare system includes 15 primary care clinics and an acute care hospital and serves a multiethnic, low-income, and under-studied population residing in various San Francisco neighborhoods. The hospital microbiology laboratory conducts all laboratory testing for the entire system. Bacteriuria episodes, representing either urinary tract infection or asymptomatic bacteriuria, were defined as a single urine culture growing *E. coli* on a unique date. If multiple cultures were sent on the same day, only one culture was considered, to avoid artificially inflating the number of bacteriuria episodes. Urine cultures from separate days were considered to be separate episodes, as we could not differentiate between an untreated and a repeat infection. Recurrent bacteriuria episodes were defined as > = 2 *E. coli* bacteriuria episodes in the last 6 months or > = 3 in the last year [21]. For this analysis, we included community-onset *E. coli* bacteriuria episodes only, defined as cases in which a urine culture, obtained in (a) an outpatient clinic or emergency department setting, or (b) within 48 h of inpatient admission, grew *E. coli*. We excluded patients who were hospitalized within 90 days of the urine culture or whose urine was sent for culture 48 h after inpatient admission. We excluded patients with a documented residence outside of San Francisco County or those living at the public skilled nursing facility. This study was approved by institutional review boards from UCSF and SFGH (IRB number 19-27233).

### Data collection

Data were extracted from electronic health records (EHR) for all *E. coli* bacteriuria cases identified from January 2014 to March 2020 by the UCSF CTSI data abstraction services. We extracted data on the urine culture organism as well as antimicrobial susceptibility testing (AST). The microbiology laboratory performs AST with Microscan and disk diffusion tests, reporting resistance based on CLSI breakpoint standards [22]. The microbiology laboratory reports ESBL-*E. coli* as an *E. coli* strain resistant to ceftazidime or cefotaxime and inhibited by clavulanic acid using broth microdilution, per 2016 CLSI guidelines [22] Bacteriuria episode caused by ESBL-*E. coli* was the main outcome of interest. Other variables included age at time of culture (0–17, 18–34, 35–64, or over 65 years); gender (women or men); self-identified race and ethnicity (American Indian or Alaska Native, Asian American, Black or African American, Latine, Native Hawaiian or other Pacific Islander, Other, or White); preferred language (any Chinese dialect, English, Other, Russian, Spanish, Tagalog, or Vietnamese); and insurance type (commercial, public, and other/unknown). Patients were considered as Latine if they identified having Latine ethnicity regardless of specified race. While analyses did not focus on differences by race and ethnicity, we conceptualize race and ethnicity as a marker of differential environmental exposures driven by cultural mores such as diet and travel.

### Geocoding and spatial analyses

As part of system-wide efforts, addresses listed in the EHR in 2019 of all patients receiving care in the public healthcare system were geocoded to latitude and longitude coordinates using ArcGIS Business Analyst 2016 (ESRI) [23]. We conducted global spatial autocorrelation analyses (Global Moran’s I) to identify spatial clusters of ESBL-*E. coli* bacteriuria episodes versus non-ESBL-*E. coli* bacteriuria episodes at multiple levels: (1) episodes and (2) individuals. Here, we define spatial clusters of ESBL-*E. coli* bacteriuria episodes as clusters of all infections, including repeat and recurrent infections, in the same individual or in individuals living near one another as a result of repeat common-source exposures. Spatial clusters of individuals with any ESBL-*E. coli* bacteriuria episodes during the study period would represent clusters of any such infection in different individuals living near one another or at the same residential address due to common-source exposures within a community.

Sensitivity analyses were conducted separately for study periods 2014–2016 and 2017–2020 to identify possible differences in spatial clustering given changes in guidelines for ESBL identification in 2016 [24]. In additional sensitivity analyses, *E. coli* bacteriuria episodes in the same individual within the same month were treated as the same episode. Analyses at the individual level were also stratified by race and ethnicity (Asian American, Black or African American, Latine, or White) and preferred language (any Chinese dialect, English, or Spanish) to identify potential differences in exposure due to patient sociodemographic characteristics. The parameters for Global Moran’s I included Euclidean distance, inverse distance for conceptualization of spatial relationships, and no standardization of spatial weights. When global clusters were identified, local clusters were assessed with Local Moran’s I, with inverse distance bands, to understand their contribution to the global clustering statistic. We applied a false discovery rate (FDR) correction which adjusts the p-value to control for multiple testing and spatial dependence [25, 26]. When multiple tests are performed on the same set of data, the probability of obtaining at least one false positive result increases. The FDR correction addresses this by controlling for the expected proportion of false positives among all the statistically significant results. This is done by calculating a critical p-value for each test with the Benjamini-Hochberg procedure. This method produces an adjusted p-value by taking the original p-value, multiplying it by the number of tests, and then dividing by the rank of that p-value. All spatial analyses were conducted using ArcMap 10.7.1 (ESRI).

### Statistical data analysis

Descriptive statistics, including frequencies and percentages for categorical data, were used to summarize variables. The likelihood of recurrent bacteriuria, (1) overall and (2) with ESBL-*E. coli* vs. non-ESBL-*E. coli*, for individuals with an initial ESBL-*E. coli* bacteriuria episode vs. non-ESBL-*E. coli* bacteriuria episode was assessed by Poisson regression models, adjusting for age category, gender, race and ethnicity, and use of antibiotics in the last 6 months. Analyses were conducted by RStudio4 version 4.0.4. We report 95% confidence intervals to characterize uncertainty in our effect estimates.

## Results

### Characteristics of the study samples and patients

From January 2014 to March 2020, 82,800 urine samples were processed at the clinical microbiology laboratory. Of these, 13,522 urine cultures grew an identifiable organism. *E. coli* was identified in 9,028 (67%) isolates (7,751 community-onset, 1,277 healthcare-onset/associated). There were 6,291 unique patients with an *E. coli* bacteriuria episode. Of the 5,576 patients who met the definition of a community-onset *E. coli* bacteriuria, 4,304 had a valid San Francisco address. Our analyses include 5,938 community-onset *E. coli* bacteriuria episodes from those 4,304 unique patients with a San Francisco address. Most patients were between the ages of 35 and 64 (46%) (Table 1). Patients with bacteriuria were primarily women (87%). The study population was multiethnic, with 46% Latine patients, 20% Asian or Asian American patients, 14% White patients, 13% Black patients, 2% Native Hawaiian or other Pacific Islander patients, and 1% American Indian or Alaska Native patients. While most patients spoke English (53%), over a third spoke Spanish (34%). Many patients had public health insurance (Medicare, Medi-Cal, or Healthy San Francisco; 43%), however, most did not have insurance information or had other insurance (56%). Supplemental Table 1 summarizes patient demographic characteristics for all 5,938 community-onset *E. coli* bacteriuria episodes.

**Table 1:** Demographic characteristics of unique patients with community-onset *E. coli* bacteriuria episodes, San Francisco, 2014–2020

	Number of patients N (%)
Age category (years)	
0–17	270 (6)
18–34	1167 (27)
35–64	1983 (46)
65+	884 (21)
Gender	
Women	3763 (87)
Men	541 (13)
Race and ethnicity	
American Indian or Alaska Native	22 (1)
Asian American	875 (20)
Black or African American	544 (13)
Latine	1967 (46)
Native Hawaiian or other Pacific Islander	70 (2)
Other	206 (5)
White	620 (14)
Preferred language	
Chinese dialect	355 (8)
English	2301 (53)
Other	75 (2)
Russian	40 (9)
Spanish	1456 (34)
Tagalog	37 (1)
Vietnamese	40 (1)
Insurance type	
Commercial	27 (1)
Public	1856 (43)
Other/Unknown	2421 (56)
Years	
2014	644 (15)
2015	624 (15)
2016	659 (15)
2017	748 (17)
2018	746 (17)
2019	719 (17)
2020	164 (4)
Total	4304

Note: Data from a public healthcare system including inpatient and outpatient services. Patients included have documented residences in San Francisco. Data obtained from January 2014 to March 2020

### Presence of spatial clusters of ESBL-*E. coli*

**Fig. 1:**
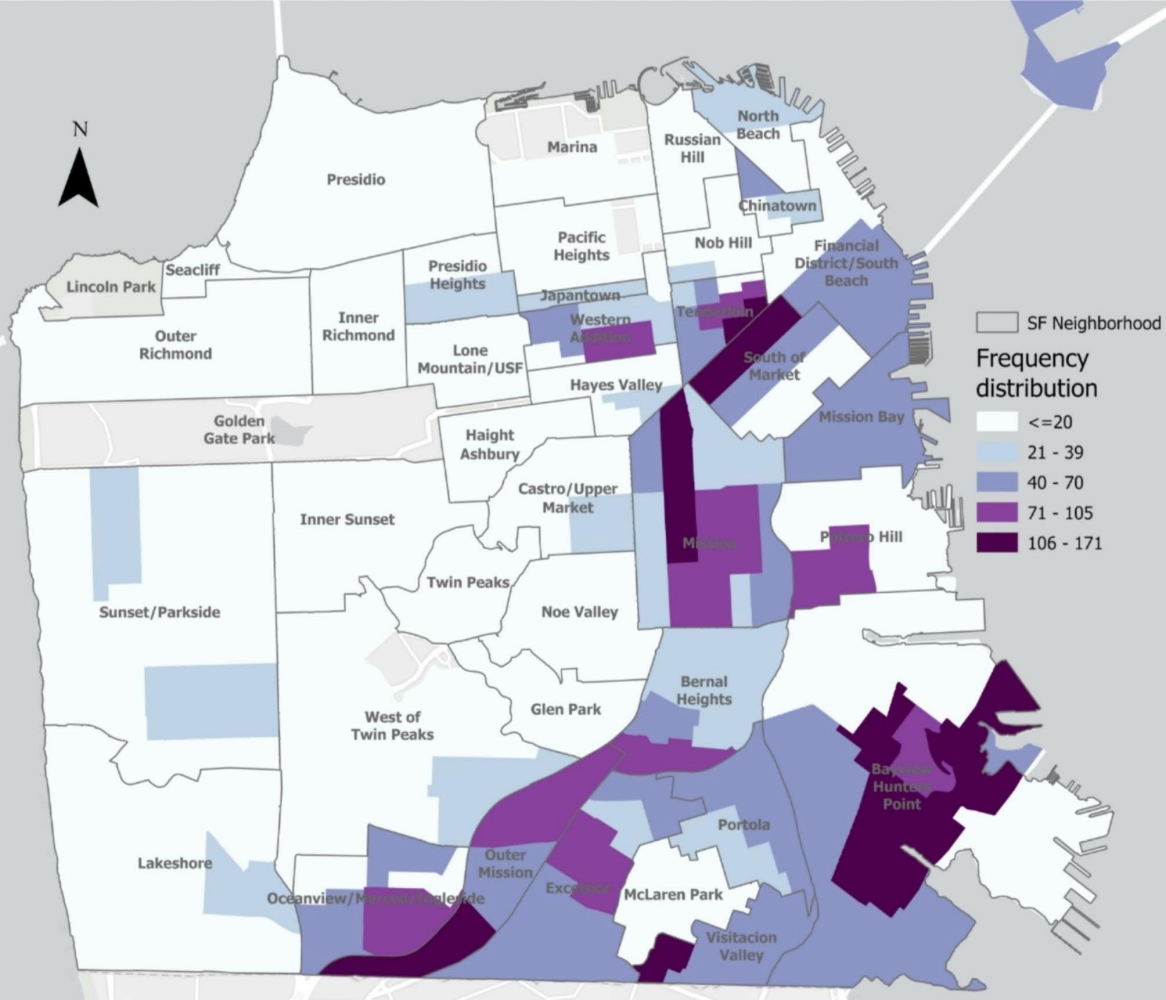
Spatial distribution of residences of individuals with *E. coli* bacteriuria episodes, San Francisco, 2014–2020 Note: Data from a public healthcare system including inpatient and outpatient services. *E. coli* bacteriuria episode from patients with documented San Francisco residence. Frequency of *E. coli* bacteriuria episodes mapped, omitting those that occurred within the same month in the same individual

**Fig. 2:**
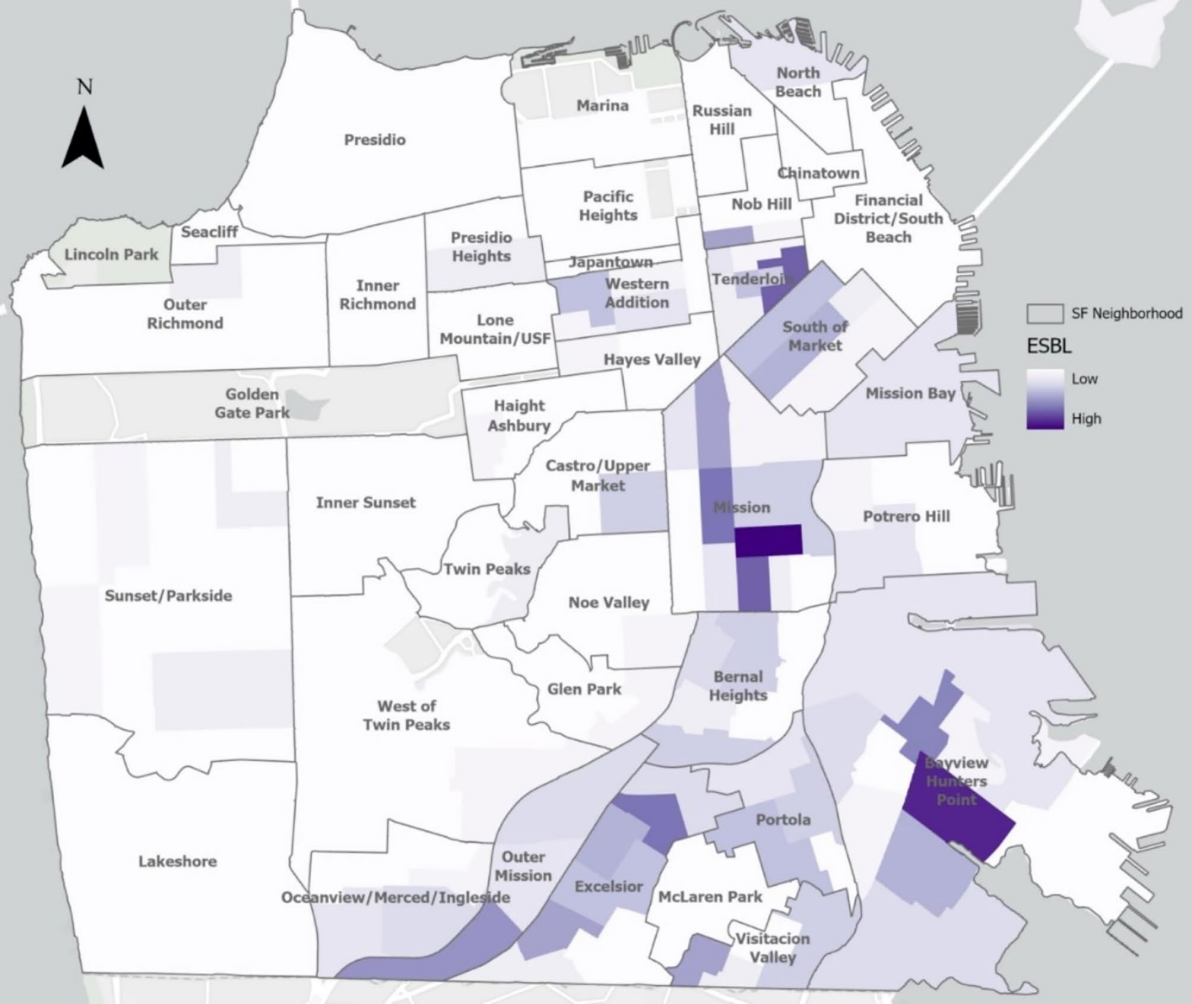
Spatial distribution of residences of individuals with ESBL-*E. coli* bacteriuria episodes, San Francisco, 2014–2020 Note: Data from a public healthcare system including inpatient and outpatient services. *E. coli* bacteriuria episode from patients with documented San Francisco residence. Due to low numbers of ESBL-*E. coli* bacteriuria episodes per neighborhood, actual numbers of episodes are not specified. All ESBL-*E. coli* bacteriuria episodes mapped, omitting those that occurred within the same month in the same individual

All 5,938 *E. coli* bacteriuria episodes from 2014 to 2020, including those that were repeat or recurrent episodes in the same patient, were mapped using patient’s geocoded residential addresses. Figure 1 shows the geographic distribution of individuals with *E. coli* bacteriuria episodes. Figure 2 shows the relative prevalence of individuals with ESBL-*E. coli* bacteriuria by San Francisco neighborhood. In Global Moran’s I models, we found evidence of spatial autocorrelation of ESBL-*E. coli* bacteriuria episodes, or spatial clusters (p < 0.001), compared to non-ESBL-*E. coli* episodes. In Local Moran’s I models, with inverse distance band, we found evidence of local spatial autocorrelation of ESBL-*E. coli* bacteriuria episodes (high-high clusters) in the neighborhoods of SOMA/Tenderloin, the Mission, and Excelsior. Sensitivity analyses excluding bacteriuria episodes within the same month also identified global and local spatial clusters (p < 0.001), even when correcting with false discovery rate.

Using the same parameters, we did not identify spatial clusters of unique individuals with any ESBL-*E. coli* bacteriuria episode vs. unique individuals with only non-ESBL-*E. coli* bacteriuria episodes (p = 0.43), meaning that there was no clustering between different individuals with any ESBL-*E. coli* bacteriuria episodes. We did not identify such spatial clusters of individuals with any ESBL-*E. coli* bacteriuria vs. only non-ESBL-*E. coli* in analyses stratified by racial and ethnic groups (Asian American patients, p = 0.8; Black patients, p = 0.3; Latine patients, p = 0.7; White patients, p = 0.98) or preferred language (Chinese dialect, p = 0.9; English, p = 0.8; Spanish, p = 0.7). Sensitivity analyses for years 2014–2016 and 2017–2020 showed no spatial clusters. We restricted data to densely populated neighborhoods, where many patients receiving care within this healthcare system live and where evidence of spatial clustering had been found when mapping all episodes. No evidence of spatial clustering of individuals with any ESBL-*E. coli* bacteriuria vs. individuals with any non-ESBL-*E. coli* bacteriuria for each neighborhood separately was identified (SOMA/Tenderloin, p = 0.7, Mission, p = 0.7, Bayview, p = 0.2, Excelsior, p = 0.3).

Spatial clusters of individuals with recurrent ESBL-*E. coli* bacteriuria episodes were identified (p < 0.0001) vs. those with recurrent non-ESBL bacteriuria episodes and single bacteriuria episodes.

### Prevalence of recurrent episode by ESBL production

Of all 5,938 *E. coli* bacteriuria episodes, 220 (4%) represented recurrent episodes (Table 2). Forty (18%) episodes were caused by ESBL-*E. coli* bacteriuria, compared to 421 (7%) non-recurrent episodes caused by ESBL-*E. coli* bacteriuria. Twenty-three (58%) recurrent episodes caused by ESBL-*E. coli* occurred after an initial ESBL-*E. coli* bacteriuria episode, compared to 8 (4%) recurrent episodes caused by ESBL-*E. coli* that occurred after an initial non-ESBL-*E. coli* bacteriuria episode. In Poisson regression models, recurrence was more likely to occur with ESBL-*E. coli* than non-ESBL *E. coli* (odds ratio [OR] 2.78, 95% confidence interval [95% CI] 2.10, 3.66, p < 0.001) and initial ESBL-*E. coli* bacteriuria episodes as part of recurrent episodes were more likely to be followed by ESBL-*E. coli* (OR 2.27, 95% CI 1.82, 2.83, p < 0.001). Moreover, with Asian American patients as the reference group, Black or African American patients (OR 2.60, 95% CI 1.31, 5.16, p < 0.01) and White patients (OR 3.90, 95% CI 1.29, 7.62, p < 0.001) had increased odds of recurrent bacteriuria episodes. Latine patients (OR 2.67, 95% CI 1.88, 3.78, p < 0.001), White patients (OR 1.56, 95% CI 1.03, 2.37, p = 0.04), and patients identifying as other (OR 2.20, 95% CI 1.36, 3.55, p < 0.001) had greater odds of having a recurrent bacteriuria episode caused by ESBL-*E. coli*.

**Table 2:** Frequency of recurrent *E. coli* bacteriuria episodes and ESBL-production

**Frequency of recurrent** ***E. coli*** **bacteriuria episodes by ESBL production**			
	Number of *E. coli* bacteriuria episodes (%)		
	Recurrent episodes	Non-recurrent episodes	Total episodes
ESBL-*E. coli* bacteriuria episode	40 (18)	421 (7)	461 (8)
Non-ESBL-*E. coli* bacteriuria episode	180 (82)	5297 (93)	5477 (92)
Total episodes	220 (100)	5718 (100)	5938 (100)
**Frequency of ESBL production in recurrent** ***E. coli*** **bacteriuria episodes by initial episode type**			
	Number of recurrent *E. coli* bacteriuria episodes (%)		
	With ESBL-*E. coli*	With non-ESBL-*E. coli*	Total episodes
Initial ESBL-*E. coli* bacteriuria episode	23 (Col% 74; Row% 58)	17 (Col% 9; Row% 42)	40 (18)
Initial non-ESBL-*E. coli* bacteriuria episode	8 (Col% 26; Row% 4)	172 (Col% 91; Row% 96)	180 (82)
Total episodes	31 (Col% 100; Row% 14)	189 (Col%100; Row% 86)	220 (100)

Note: Data from a public healthcare system including inpatient and outpatient services. *E. coli* bacteriuria episode from patients with documented San Francisco residence

## Discussion

To our knowledge, this is the first report identifying spatial clusters of community-onset ESBL-*E. coli* causing bacteriuria. We detected evidence of spatial clusters of ESBL-*E. coli* bacteriuria episodes within a large urban safety net healthcare system, with data spanning 6 years. Such clusters were not replicated when mapping unique individuals with any ESBL-*E. coli* bacteriuria episode during the study period. In further analyses, we found that patients with an initial ESBL-*E. coli* bacteriuria episode were more likely to have recurrent bacteriuria episodes, particularly caused by ESBL-*E. coli*. Thus, the spatial clusters of ESBL-*E. coli* bacteriuria episodes may be explained by bacteriuria recurrence in the same patients. This suggests the lack of evidence of local geographic *E. coli* sources affecting multiple individuals. However, it may be that certain individuals may be exposed to the same *E. coli* sources, leading to recurrent bacteriuria episodes.

Few reports to date have assessed the presence of spatial clusters of infections caused by ESBL-producing Enterobacteriaceae and all have used different methods to report such clusters. In a 2-year study based on community-onset infections, Sarda et al. found spatial clusters of ceftriaxone-resistant Enterobacteriaceae isolates by census tracts from a single healthcare center in Cook County, Illinois [20]. Patients living in census tracts with a higher percentage of Latine, foreign-born, and uninsured residents were more likely to have an infection with ceftriaxone-resistant Enterobacteriaceae. They mapped all isolates except those that were duplicates, and 85% of the isolates came from urine. In a multi-center study, Logan et al. found that children living in South Chicago and those who were diagnosed in outpatient settings were more likely to have infections with CTX-M-9-group-producing Enterobacteriaceae [27]. Arias Ramos et al. mapped ESBL-producing Enterobacteriaceae isolates from community infections and colonization events diagnosed at an urban center in Colombia [28]. Using kernel density estimations, they found hotspots of patients with community-acquired infections caused by ESBL-producing Enterobacteriaceae in various communes. Galvin et al. also found spatial clusters of drug-resistant *E. coli* causing urinary tract infections in urban areas in the West of Ireland [17] Yet, most of these studies did not assess whether spatial clustering was driven by repeat or recurrent infections in the same individuals.

There are multiple factors that could explain spatial clustering of drug-resistant organisms. First, geographical patterns in antibiotic prescribing or consumption, at the neighborhood-level, may drive selection of drug-resistant *E. coli*, as suggested by studies finding infection or colonization with fluoroquinolone-resistant *E. coli* to be associated with neighborhood-level fluoroquinolone consumption [18, 29, 30]. Second, environmental exposures to such organisms may drive community transmission, as noted by Arias Ramos et al., where communes with higher prevalence of infections caused by ESBL-producing *Enterobacteriaceae* were located close to rivers and hospitals [28] Third, as we have found, it may be that spatial clusters of drug-resistant infections are explained by recurrent infections. In our analyses, we found that patients with an initial community-onset ESBL-*E. coli* bacteriuria episode were more likely to have recurrent episodes, particularly with ESBL-*E. coli*. While others have reported such recurrence patterns, they have not focused on community-onset infections [31, 32].

When we conducted sub-analyses by race and ethnicity and preferred language, given historical segregation and the presence of ethnic enclaves in San Francisco, we found no evidence of spatial clusters of individuals with any ESBL-*E. coli* bacteriuria episode [32]. Sub-analyses by time period, as well as those restricted to densely-populated neighborhoods, showed no evidence of clusters. The initial spatial clusters of ESBL-*E. coli* bacteriuria episodes we found may be entirely explained by recurrent ESBL-*E. coli* bacteriuria. Yet, it is also feasible that ESBL-*E. coli* bacteriuria episodes are caused by a variety of *E. coli* genotypes representative of multiple common source exposures throughout San Francisco. Mapping specific genotypes, as opposed to drug-resistant phenotypes, would help elucidate this hypothesis. Relatedly, Nobrega et al. reported spatial clusters of bloodstream infections caused by ST131-C2 subclade in a North East Calgary sector without long-term care facilities [19]. Exposure to food and travel has been found to be important drivers of transmission of drug-resistant *E. coli*. [13, 34, 35] Thus, it may be that, alternatively, foodborne outbreaks of ESBL-*E. coli* did not occur in spatially distinct patterns given the widespread food distribution in metropolitan areas through large supermarket chains.

Although this is the first multi-year study evaluating the presence of spatial clusters of community-onset ESBL-*E. coli* bacteriuria from a large urban healthcare system caring for diverse populations, our study has several limitations. First, we report data from one healthcare system, which limited our sample size and may have resulted in selection bias. Future studies may include other healthcare systems in San Francisco to better identify spatial clusters involving the entire prevalence of uropathogenic ESBL-*E. coli* in San Francisco. Second, we did not have access to pathogen genotype data, as it is not routinely collected for clinical management purposes, and thus we could not assess spatial patterns of specific genotypes producing ESBL. We did not conduct spatial clusters analyses of antibiotic susceptibility phenotypes as a proxy for genotype for the following reasons: (1) genotypes may harbor or lose mobile genetic elements which may change antibiotic susceptibility phenotype and (2) our study was not powered to assess spatial clustering of the various antibiotic susceptibility phenotypes. Third, we used patients’ last known geocoded address as of 2019, which may have resulted in some geographic misclassification. This may have led to a small margin of error, as about 10% of the population within the most represented neighborhoods in our study moved within the same county, and about 5% moved from a different county throughout the study period [36]. Fourth, we were not able to assess seasonal changes in ESBL-*E. coli* spatial prevalence due to sample size. Indeed, sensitivity analyses conducted for years 2014–2016 and 2017–2020 did not identify any ESBL-*E. coli* bacteriuria episode spatial clusters. Finally, our data, as any other healthcare system-based data, were limited to individuals who had a processed urine culture. However, current IDSA guidelines do not recommend routine urine culture testing in all patients with symptoms suggesting urinary tract infection [37]. As such, data on all patients with a community-onset urinary tract infection would not be available in any other healthcare system-based study.

## Conclusion

In analyses spanning 6 years at a large urban safety-net healthcare system, we found spatial clustering of ESBL-*E. coli* bacteriuria episodes among patients in community settings. However, analyses among unique individual did not show such clusters, suggesting that the clusters we initially found may be explained by the recurrence of ESBL-*E. coli* bacteriuria in the same individuals. These findings have important implications in understanding the epidemiology of ESBL-*E. coli* and in the clinical treatment of patients with past ESBL-*E. coli* bacteriuria, such as the greater odds of recurrent bacteriuria after an initial episode caused by ESBL-*E. coli*. Future work will include identification of spatial clusters of genotypes as such level of information would be more adequate markers of common-source exposures.

### Electronic supplementary material

Below is the link to the electronic supplementary material.

Supplementary Material 1

## Data Availability

The datasets used and/or analyzed during the current study are available from the corresponding author on reasonable request.

## References

[1] CDC (2019). *Antibiotic resistance threats in the United States, 2019* Atlanta.

[2] Kassakian SZ, Mermel LA (2014). Changing epidemiology of Infections due to extended spectrum beta-lactamase producing bacteria. Antimicrob Resist Infect Control.

[3] van Driel AA, Notermans DW, Meima A et al. Antibiotic resistance of Escherichia coli isolated from uncomplicated UTI in general practice patients over a 10-year period. Eur J Clin Microbiol Infect Dis 2019. 10.1007/s10096-019-03655-3 PMC6800841 31440915

[4] Pitout JD, Laupland KB (2008). Extended-spectrum beta-lactamase-producing Enterobacteriaceae: an emerging public-health concern. Lancet Infect Dis.

[5] Bours PH, Polak R, Hoepelman AI, Delgado E, Jarquin A, Matute AJ (2010). Increasing resistance in community-acquired urinary tract Infections in Latin America, five years after the implementation of national therapeutic guidelines. Int J Infect Dis.

[6] Lagunas-Rangel FA (2018). Antimicrobial susceptibility profiles of bacteria causing urinary tract Infections in Mexico: single-centre experience with 10 years of results. J Glob Antimicrob Resist.

[7] Park JJ, Seo YB, Lee J (2017). Antimicrobial susceptibilities of Enterobacteriaceae in Community-acquired urinary tract Infections during a 5-year period: a single hospital study in Korea. Infect Chemother.

[8] Doi Y, Park YS, Rivera JI (2013). Community-associated extended-spectrum beta-lactamase-producing Escherichia coli Infection in the United States. Clin Infect Dis.

[9] de Souza da-Silva AP, de Sousa VS, de Araújo Longo LG (2020). Prevalence of fluoroquinolone-resistant and broad-spectrum cephalosporin-resistant community-acquired urinary tract Infections in Rio De Janeiro: impact of genotypes ST69 and ST131. Infect Genet Evol.

[10] Butcher CR, Rubin J, Mussio K, Riley LW (2019). Risk factors Associated with Community-acquired urinary tract Infections caused by extended-spectrum ≤-Lactamase-producing Escherichia coli: a systematic review. Curr Epidemiol Rep.

[11] Soraas A, Sundsfjord A, Sandven I, Brunborg C, Jenum PA (2013). Risk factors for community-acquired urinary tract Infections caused by ESBL-producing enterobacteriaceae–a case-control study in a low prevalence country. PLoS ONE.

[12] Strysko JP, Mony V, Cleveland J, Siddiqui H, Homel P, Gagliardo C (2016). International travel is a risk factor for extended-spectrum β-lactamase-producing Enterobacteriaceae acquisition in children: a case-case-control study in an urban U.S. hospital. Travel Med Infect Dis.

[13] Ukah UV, Glass M, Avery B (2018). Risk factors for acquisition of multidrug-resistant Escherichia coli and development of community-acquired urinary tract Infections. Epidemiol Infect.

[14] Nordstrom L, Liu CM, Price LB (2013). Foodborne urinary tract Infections: a new paradigm for antimicrobial-resistant foodborne Illness. Front Microbiol.

[15] Riley LW (2020). Extraintestinal Foodborne pathogens. Annu Rev Food Sci Technol.

[16] CDC (2012). Principles of Epidemiology in Public Health Practice: an introduction to Applied Epidemiology and Biostatistics.

[17] Galvin S, Bergin N, Hennessy R (2013). Exploratory spatial mapping of the occurrence of Antimicrobial Resistance in E. Coli in the community. Antibiot (Basel).

[18] Kiffer CR, Camargo EC, Shimakura SE (2011). A spatial approach for the epidemiology of antibiotic use and resistance in community-based studies: the emergence of urban clusters of Escherichia coli quinolone resistance in Sao Paulo, Brasil. Int J Health Geogr.

[19] Nobrega D, Peirano G, Lynch T, Finn TJ, Devinney R, Pitout JDD (2021). Spatial distribution of Escherichia coli ST131 C subclades in a centralized Canadian urban region. J Antimicrob Chemother.

[20] Sarda V, Trick WE, Zhang H, Schwartz DN (2021). Spatial, ecologic, and clinical epidemiology of Community-Onset, Ceftriaxone-Resistant Enterobacteriaceae, Cook County, Illinois, USA. Emerg Infect Dis.

[21] Aydin A, Ahmed K, Zaman I, Khan MS, Dasgupta P (2015). Recurrent urinary tract Infections in women. Int Urogynecol J.

[22] CLSI. *Performance Standards for Antimicrobial Susceptibility Testing* Wayne, PA2016.

[23] Liu EF, Rubinsky AD, Pacca L (2022). Examining Neighborhood Socioeconomic Status as a mediator of Racial/Ethnic disparities in Hypertension Control Across Two San Francisco Health Systems. Circ Cardiovasc Qual Outcomes.

[24] Poulou A, Grivakou E, Vrioni G (2014). Modified CLSI extended-spectrum beta-lactamase (ESBL) confirmatory test for phenotypic detection of ESBLs among Enterobacteriaceae producing various beta-lactamases. J Clin Microbiol.

[25] Benjamini Y, Hochberg Y (1995). Controlling the false discovery rate: a practical and powerful approach to multiple testing. J Roy Stat Soc: Ser B (Methodol).

[26] Caldas de Castro M, Singer BH (2006). Controlling the false Discovery rate: a New Application to Account for multiple and dependent test in Local Statistics of Spatial Association. Geographical Anal.

[27] Logan LK, Medernach RL, Domitrovic TN (2019). The clinical and molecular epidemiology of CTX-M-9 Group Producing Enterobacteriaceae Infections in Children. Infect Dis Ther.

[28] Arias Ramos D, Hoyos Pulgarin JA, Moreno Gomez GA (2020). Geographic mapping of Enterobacteriaceae with extended-spectrum beta-lactamase (ESBL) phenotype in Pereira, Colombia. BMC Infect Dis.

[29] Terahara F, Nishiura H (2019). Fluoroquinolone consumption and Escherichia coli resistance in Japan: an ecological study. BMC Public Health.

[30] Low M, Neuberger A, Hooton TM (2019). Association between urinary community-acquired fluoroquinolone-resistant Escherichia coli and neighbourhood antibiotic consumption: a population-based case-control study. Lancet Infect Dis.

[31] Ahn ST, Kim SW, Kim JW, Park HS, Moon DG, Oh MM (2019). Does urinary tract Infection caused by extended-spectrum beta-lactamase-producing Escherichia coli show same antibiotic resistance when it recurs?. J Infect Chemother.

[32] Lindblom A, Kiszakiewicz C, Kristiansson E (2022). The impact of the ST131 clone on recurrent ESBL-producing E. Coli urinary tract Infection: a prospective comparative study. Sci Rep.

[33] Menendian S, Gambhir S (2018). Racial Segregation in the San Francisco Bay Area.

[34] Doi Y, Iovleva A, Bonomo RA (2017). The ecology of extended-spectrum beta-lactamases (ESBLs) in the developed world. J Travel Med.

[35] Liu CM, Stegger M, Aziz M (2018). Escherichia coli ST131-*H*22 as a Foodborne Uropathogen. mBio.

[36] USCB. S0701 Geographic mobility by selected characteristics in the United States. In: ACS; 2021.

[37] Gupta K, Hooton TM, Naber KG (2011). International clinical practice guidelines for the treatment of acute uncomplicated cystitis and pyelonephritis in women: a 2010 update by the Infectious Diseases Society of America and the European Society for Microbiology and Infectious Diseases. Clin Infect Dis.

