# Supplementary material for: Spatial clusters of extended-spectrum beta-lactamase-producing Escherichia coli causing community-onset bacteriuria due to repeat infections: cluster analysis from a large urban medical center, San Francisco, 2014–2020
Source: Antimicrob Resist Infect Control. 2023 Oct 20;12:115. doi: 10.1186/s13756-023-01320-1 (PMC10588154; doi:10.1186/s13756-023-01320-1)
Supplement: Supplementary file 1 — Supplementary Material 1 [file 13756_2023_1320_MOESM1_ESM.docx]

Supplemental table 1. Demographic characteristics of patients with community-onset *E. coli* bacteriuria episodes, San Francisco, 2014-2020

|  | Number of episodes  N (%) |
| --- | --- |
| Age category (years) |  |
| 0-17 | 325 (5) |
| 18-34 | 1502 (26) |
| 35-64 | 2717 (46) |
| 65+ | 1394 (23) |
| Sex |  |
| Women | 5234 (88) |
| Men | 704 (12) |
| Race and ethnicity |  |
| American Indian or Alaska Native | 31 (<1) |
| Asian American | 1154 (19) |
| Black or African American | 729 (12) |
| Latine | 2825 (48) |
| Native Hawaiian or other Pacific Islander | 98 (2) |
| Other | 273 (5) |
| White | 828 (14) |
| Preferred language |  |
| Chinese dialect | 479 (8) |
| English | 3112 (52) |
| Other | 259 (4) |
| Spanish | 2088 (35) |
| Insurance type |  |
| Commercial | 38 (<1) |
| Public | 3037 (51) |
| Other/Unknown | 2901 (49) |
| Years |  |
| 2014 | 838 (14) |
| 2015 | 863 (14) |
| 2016 | 928 (16) |
| 2017 | 1023 (17) |
| 2018 | 1036 (17) |
| 2019 | 1024 (17) |
| 2020 | 226 (4) |
| Total | 5938 |
|  |  |

Note: Data from a public healthcare system including inpatient and outpatient services. Patients

included have documented residences in San Francisco
